# Neoadjuvant therapy for colorectal cancer from 2015 to 2024: a visual analysis and bibliometric analysis

**DOI:** 10.3389/fonc.2025.1526610

**Published:** 2025-04-02

**Authors:** Boyu Kang, Yihuan Qiao, Jun Zhu, Jipeng Li

**Affiliations:** ^1^ Department of Digestive Surgery, Xijing Hospital of Digestive Diseases, Air Force Military Medical University, Xi’an, China; ^2^ Department of General Surgery, The Southern Theater Air Force Hospital, Guangzhou, China; ^3^ Department of Experiment Surgery, Xijing Hospital, Fourth Military Medical University, Xi’an, China

**Keywords:** colorectal cancer, neoadjuvant therapy, Bibliometrix, CiteSpace, VOSviewer

## Abstract

**Background:**

Colorectal cancer (CRC) imposes a substantial burden on global health., but research trends and hotspots in this field are still not clear. The purpose of this research is to create a visual knowledge map based on bibliometric analysis, identify research hotspots and predict future research trends.

**Method:**

Utilizing the Web of Science Core Collection (WoSCC) as data source and integrating the visualization capabilities of the Bibliometrix R software package, CiteSpace, and VOSviewer, analyze the authors, research institutions, countries, cited documents, publishing journals, abstracts, and keyword information of literature pertaining to neoadjuvant therapy for colorectal cancer spanning from January 2015 to December 2024.

**Result:**

The analysis included 1,587 articles from 1,464 institutions, 385 journals, and 61 countries or regions. China has the largest number of publications (449) and the largest number of citations (5,035). The United States occupies the leading position with an average of 21.6. “Annals of Surgical Oncology” is the most published journal with 51 articles, and “Journal of Clinical Oncology” is the journal with the most references (4,465 references). Highly cited references focus on clinical trials and guidelines for neoadjuvant therapy for colorectal cancer. In recent years, the most important keywords in the research on colorectal cancer and neoadjuvant therapy have been “artificial intelligence”, “total neoadjuvant therapy” and “immunotherapy”.

**Conclusion:**

This article provided a review of the research on neoadjuvant therapy for colorectal cancer, can provide reference for subsequent research on neoadjuvant therapy for colorectal cancer. The results offered valuable insights and data that informed the direction of future advancements.

## 1 Introduction

Colorectal cancer(CRC) is one of the most common malignant tumors worldwide. According to the 2022 global cancer statistics, colorectal cancer has become the second leading cause of cancer-related deaths (1). Therefore, it is particularly significant to propose more optimized treatment plans for colorectal cancer. Neoadjuvant therapy refers to a preoperative anti-tumor treatment aimed at reducing the risk of recurrence increasing survival rates. Neoadjuvant radiotherapy, neoadjuvant chemotherapy and neoadjuvant immunotherapy are included. Its role continues to develop with the advancement of existing treatment methods and the improvement of neoadjuvant therapy and subsequent surgical indications (2). While improving strategies, predicting treatment outcomes through biomarkers and providing personalized treatment has become a focus of attention in recent years (3). Therefore, in the field of neoadjuvant therapy for colorectal cancer, research on neoadjuvant immunotherapy and prognostic markers related to neoadjuvant therapy will become a hot topic in future studies.

Bibliometric analysis and visualization are deemed highly valuable research methods that utilize statistical methods and visualization tools to quantify and interpret academic publications, enabling researchers to gain a comprehensive understanding of the research prospects and trends in the field during specific periods, and providing references for further research (4). The primary functions include analyzing the scientific achievements of authors, institutions, and countries within the research field, as well as predicting potential research hotspots (5).

There is currently a lack of systematic bibliometric analysis to classify and examine the prevalent research trends and hotspots in this field. To bridge this gap, we conducted a bibliometric analysis of the literature on neoadjuvant therapy for colorectal cancer published in the Web of Science Core Collection database from 2015 to 2024. Utilizing CiteSpace, VOSviewer software, and Bibliometrix R package, understand the most influential countries, journals, authors, and institutions in the field. Additionally, we sought to obtain information on hot co-cited literature, sudden co-cited literature, hot keywords, and keywords with the citation bursts. This study strives to explore the comprehensive visual knowledge graph of neoadjuvant therapy for colorectal cancer, clarify research trends, grasp hotspots and research gaps, and facilitate subsequent comprehensive and in-depth research.

## 2 Materials and methods

### 2.1 Retrieval strategy and data collection

**Figure 1:**
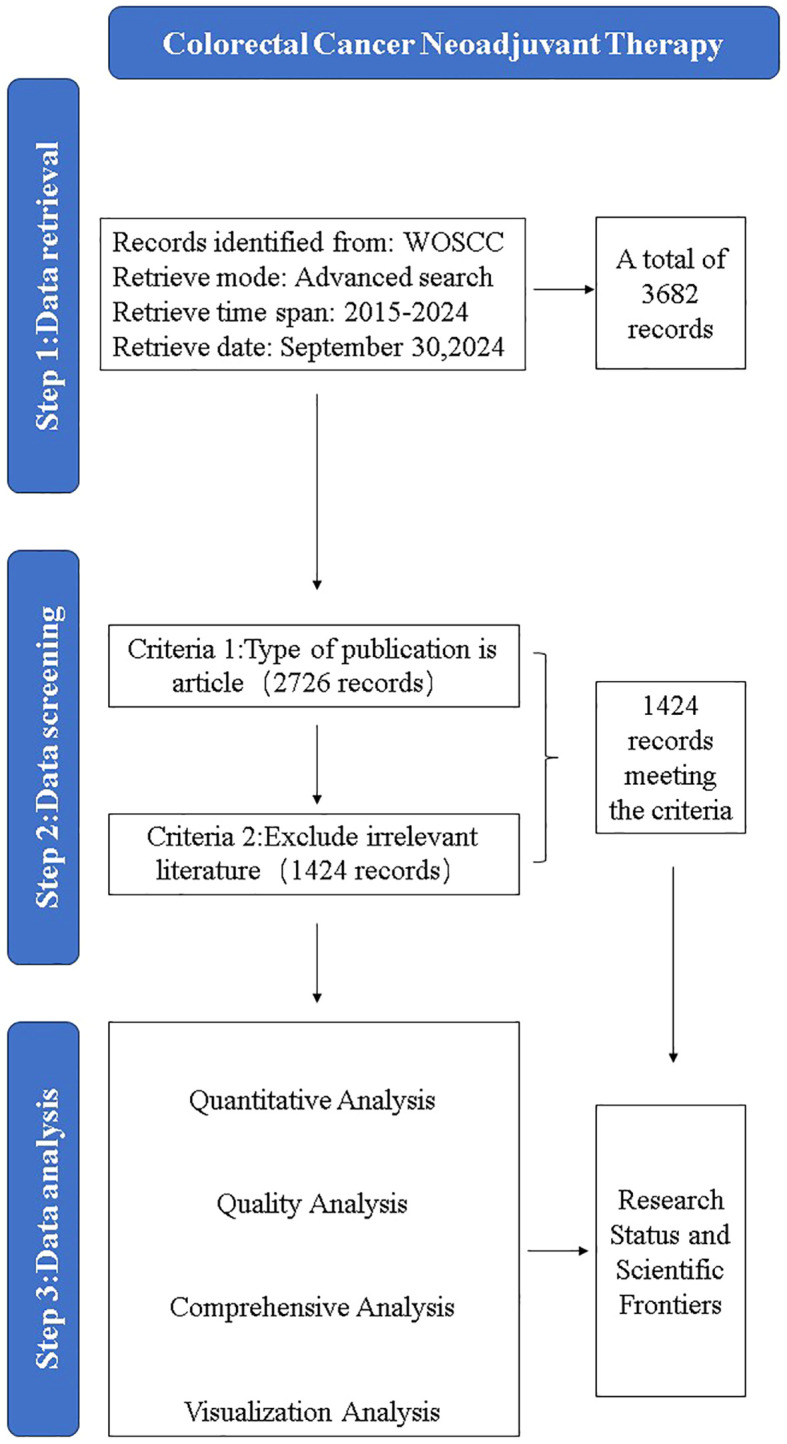
Flow chart of literature selection.

We chose Web of Science Core Collection (WoSCC) as the source database for data retrieval in this study. The data retrieval strategies are as follows: #1, ((((ts= (CRC)) or ts= (colorectal neoplasia)) or ts= (colorectal tumor)) or ts= (colorectal cancer)) or ts= (colorectal carcinoma)# 2, (((((ts= (neoadjuvant treatment)) or ts= (neoadjuvant Chemistry)) or ts= (neoadjuvant chemotherapy)) or ts= (neoadjuvant systemic therapy)) or ts= (neoadjuvant radiation)) or ts= (neoadjuvant Immunology), #1 AND #2. The search period was set to August 2024, and the material type was limited to monographs. After excluding irrelevant literatures, 1587 articles met the inclusion criteria. The flow chart of literature screening is shown in **Figure 1**.

### 2.2 Data analysis

We selected the articles published within the past 10 year. We employed bibliometric analysis algorithm to identify the references with the highest citation explosion point, keyword explosion point and citation, keyword clustering, so as to reveal the research hotspots and trends in the field of neoadjuvant therapy for colorectal cancer.

The steps of bibliometric analysis are as follows: We utilized VOSviewer 1.6.20 (Leiden University, the Netherlands), CiteSpace 6.3.R1 (Drexel University, Pennsylvania, USA) and the bibliometrix R package (4.3.0) to analyze the selected literature. The content of the analysis mainly includes countries, institutions, authors, journals, references and keywords. Data were stored and processed in txt format.

## 3 Result

### 3.1 Trends in publications of studies related to neoadjuvant therapy for colorectal cancer

**Figure 2:**
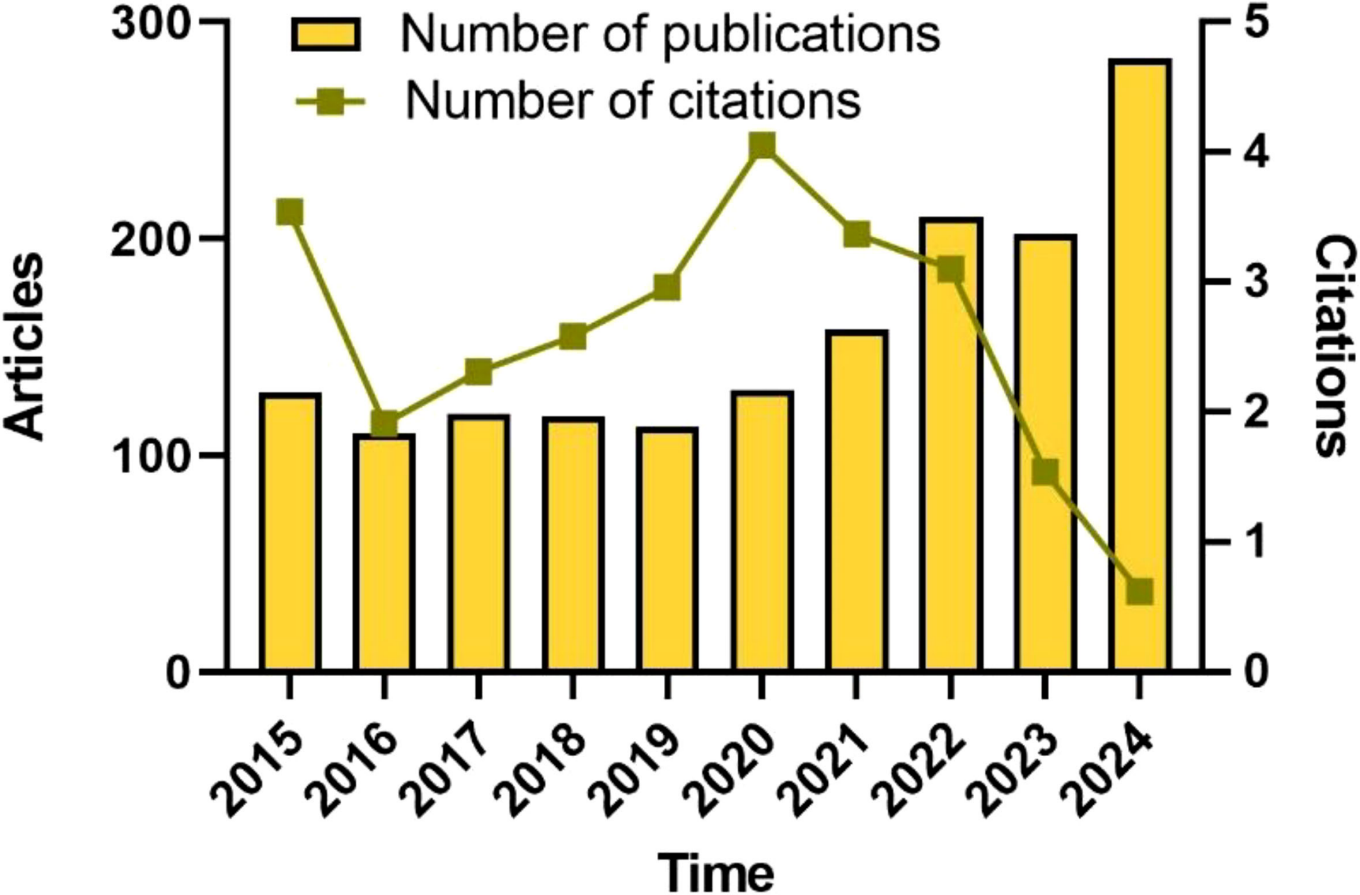
Trends in annual publications on neoadjuvant therapy for colorectal cancer.

The analysis encompassed 1,587 articles retrieved from the WoSCC database from 2015 to 2024, which were screened based on inclusion and exclusion criteria. Out of these, 32 were proceedings papers, 26 were early access articles. The total number of citations amounted to 34739. It outlines the annual publications count and citations frequency of neoadjuvant therapy for colorectal cancer, revealing a gradual yet noticeable growth trend (**Figure 2**). Over the past decade, the number of publications on neoadjuvant therapy for colorectal cancer fluctuated steadily. Despite a slight decrease in publication numbers in 2019, the average number of citations increased, indicatinga sustained interest in the field of neoadjuvant therapy for colorectal cancer. Notably, both the publication and citation counts declined in 2023 and 2024, potentially attributed to the limited time frame for data collection and the fact that many articles were still in the review stage. We analyzed the top 10 cited literatures in 2023. The results showed that, compared with other years, 2023 publications were characterized by a relatively low proportion of highly cited articles and a reduced publication count, which may be related to the standardization of neoadjuvant therapy in the guidelines and a scarcity of groundbreaking research in this domain. This also hints at the need to focus on enhancing the quality of articles in this field in the future.

### 3.2 Author output, collaboration, and citation analysis

**Figure 3:**
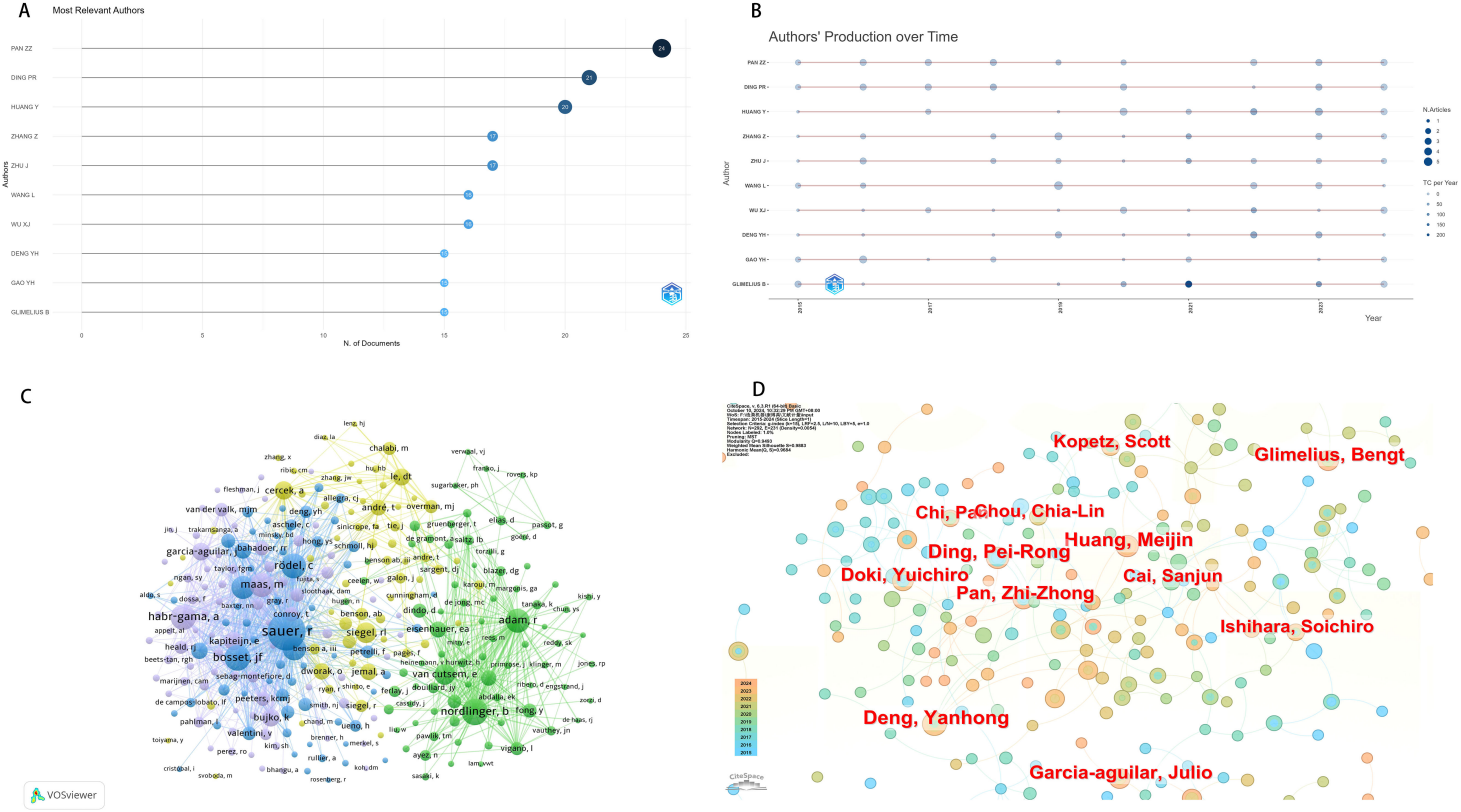
Neoadjuvant therapy for colorectal cancer. **(A)**The number of articles published by the authors. **(B)** Time chart of author output. **(C)** Co-citation chart of authors. **(D)** Author collaboration chart.

In the literature we included, a total of 12565 authors contributed to research in this field. The data reveals that from 2015 to 2024, Zhizhong Pan from Sun Yat sen University was the author with the highest total number of publications on neoadjuvant therapy for colorectal cancer(**Figure 3A**, **Table 1**). The most cited author is Rolf Sauer from Universit ä tsklinikum Erlangen, who published the CAO/ARO/AIO-04 study in Lancet Oncology in 2015, providing reference for neoadjuvant chemotherapy and adjuvant chemotherapy in locally advanced rectal cancer patients and promoting the development of neoadjuvant therapy. We further analyzed the map of author’s annual publication volume, where larger circles represent higher annual publication volumes, and darker blue hues indicate higher annual citations (**Figure 3B**). In terms of co-citation by authors, Rolf Sauer, Bernard Nordlinger, and Angelita Habr-Gama received the most citations, indicating the pivotal role of their related research in the field of neoadjuvant therapy for CRC (**Figure 3C**). In terms of collaboration, Zhizhong Pan and Peirong Ding have forged an establishing cooperative network; However, other authors generally have less collaboration (**Figure 3D**).

**Table 1:** Neoadjuvant therapy for colorectal cancer author basic information.

Author	h_index	g_index	m_index	TC	NP	PY_start
ZHANG Z	**12**	17	**1.2**	420	17	2015
DING PR	11	19	1.1	364	21	2015
GLIMELIUS B	11	15	1.1	**1382**	15	2015
PAN ZZ	11	**21**	1.1	445	**24**	2015
CAI SJ	9	12	0.9	308	12	2015
DENG YH	9	15	0.9	416	15	2015
GAO YH	9	13	0.9	184	15	2015
UENO M	9	12	0.9	276	12	2015
VERHOEF C	9	13	0.9	395	13	2015
XU Y	9	13	0.9	353	13	2015

TC, Total Citations; NP, Number of Publications; PY_start, Publication Year Start.

Bolded text indicates the maximum value in this column.

### 3.3 Country/region and institution contributions analysis

**Figure 4:**
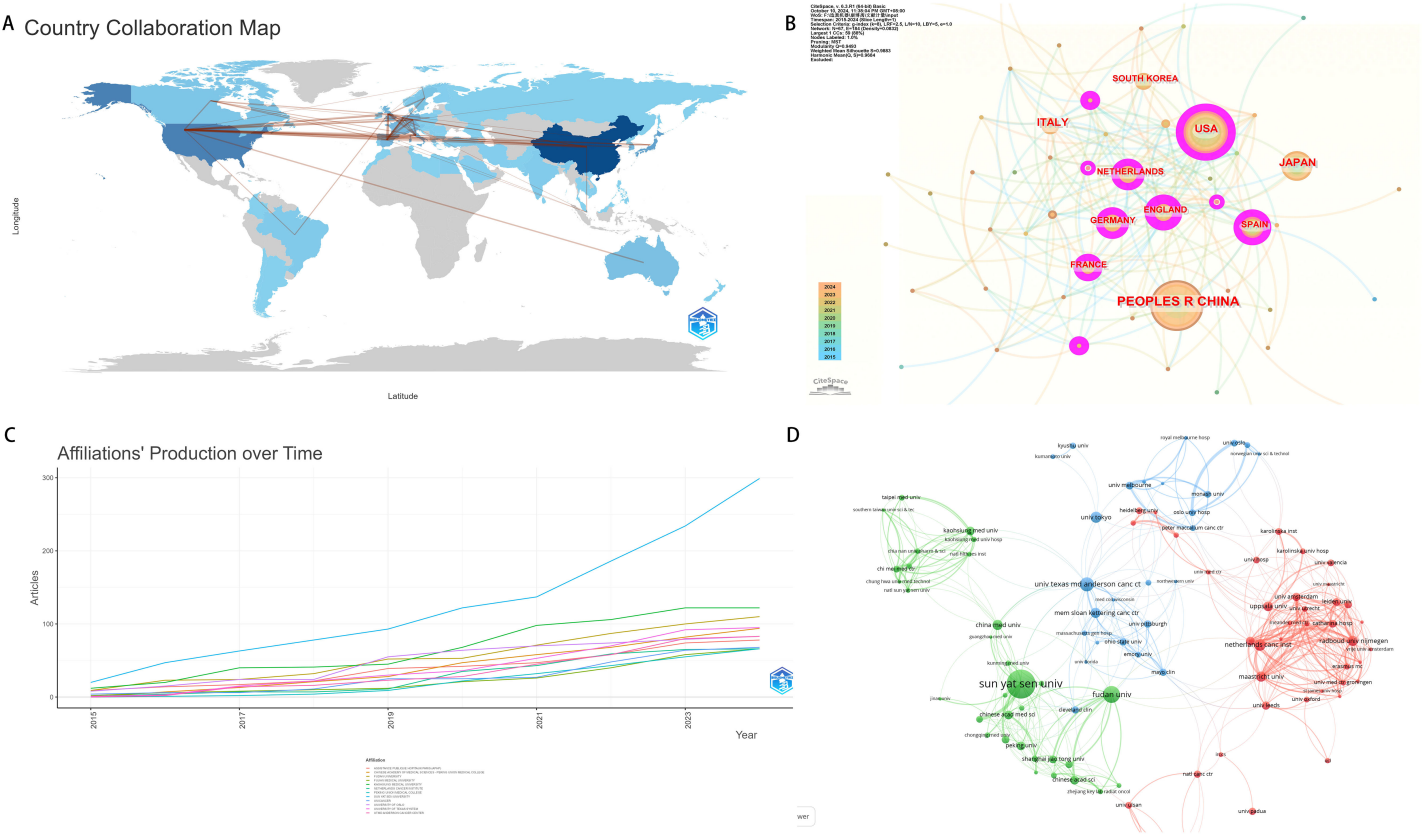
Country/region and institution contributions analysis. **(A)** National document volume map of neoadjuvant therapy for colorectal cancer. **(B)** National cooperation map. **(C)** Institutional document trend map. **(D)** Institutional interaction map.

We obtained the top 10 countries with the largest number of publications, including China, followed by the United States, Netherlands and Italy. It is particulary noteworthy that China has a small number of average cited literatures, which indicates that China has great potential for cooperation in this field in the future, but the quality of articles needs to be enhanced. Compared with other countries, the United States ranked second in the number of publications, the total number of citations, and the average number of citations, indicating that the United States has a high academic status and influence in the field of neoadjuvant therapy for colorectal cancer (**Figure 4A**). We calculated the number of papers from the top 50 countries, and used the fisher.test function of R language to test the number of papers output from developed and developing countries with the number of publications ranking in the top 50 (**Table 2**). The result indicated that the status of being a developed country did not demonstrate statistical significance in terms of the number of research papers published, which may be attributed to the relatively small sample size. However, this finding encourages researchers from economically less developed countries to pursue further studies. In the cooperation network, the United States collaborates frequently with other countries, indicating that the United States plays an crucial bridging role in the cooperation network in the field of colorectal cancer neoadjuvant therapy research, and has close ties with other countries (**Figure 4B**, **Table 3**). As of 2019, research indicates that the incidence rate of colorectal cancer in North American countries has remained at a relatively high level, which may be one of the reasons why the United States conducts more research in this field (6–8). The rapid increase in the incidence rate in Central and East Asian countries should draw the attention of countries such as China and Vietnam (9). In terms of research institutions, the top institution with published literature is Sun Yat-sen University. (**Figure 4C**). Sun Yat-sen University is at the forefront in the field of colorectal cancer research, and has consistently maintained a high level of publication output, with other institutions showing little difference in their publication volumes in this field. We found that University of Texas M.D. Anderson Cancer Center has more institutional cooperation, but other institutional cooperation is mostly limited to geographical regions. (**Figure 4D**).

**Table 2:** Number of documents published by developing and developed countries.

Developing Countries	Total Citations	Developed Countries	Total Citations
CHINA	5035	USA	4452
TURKEY	166	NETHERLANDS	4009
BRAZIL	147	ITALY	1566
INDIA	126	UNITED KINGDOM	1318
EGYPT	35	JAPAN	1284
SERBIA	35	AUSTRALIA	824
COLOMBIA	13	GERMANY	692
ARGENTINA	22	KOREA	681
RUSSIA	13	SPAIN	679
ISRAEL	12	FRANCE	649
MOROCCO	11	DENMARK	430
THAILAND	9	SWEDEN	354
PAKISTAN	8	AUSTRIA	297
ROMANIA	8	NORWAY	228
IRAN	5	BELGIUM	213
MALAYSIA	4	CANADA	207
BULGARIA	2	PORTUGAL	125
SAUDI ARABIA	2	IRELAND	92
		CZECH REPUBLIC	46
		TUNISIA	35
		NEW ZEALAND	34
		GREECE	27
		CAMBODIA	24
		FINLAND	24
		JAMAICA	24
		POLAND	24
		SWITZERLAND	24
		GEORGIA	19
		HUNGARY	17
		CROATIA	11
		LITHUANIA	9
		SINGAPORE	7
		SLOVENIA	5

**Table 3:** Centrality of national cooperation.

Country	Cluster	Betweenness	Closeness	PageRank
USA	5	**164.719**	0.016	**0.086**
UNITED KINGDOM	5	102.22	**0.017**	0.078
SPAIN	5	55.21	0.016	0.064
FRANCE	5	99.987	**0.017**	0.061
ITALY	5	21.021	0.015	0.057
GERMANY	5	34.579	0.016	0.054
NETHERLANDS	5	53.496	0.016	0.046
CHINA	5	19.222	0.014	0.044
BELGIUM	5	27.917	0.015	0.038
CANADA	3	62.688	0.015	0.033
NORWAY	5	5.28	0.014	0.026
JAPAN	5	4.64	0.013	0.026
SWEDEN	5	8.254	0.013	0.024
BRAZIL	5	5.037	0.014	0.024
KOREA	6	47.537	0.013	0.023
AUSTRALIA	**7**	80	0.013	0.022
RUSSIA	5	2.669	0.013	0.022
SINGAPORE	5	1.292	0.013	0.021
AUSTRIA	4	51.284	0.012	0.02
SLOVENIA	5	0.232	0.013	0.019
SWITZERLAND	5	3.225	0.013	0.018
PORTUGAL	3	5.483	0.013	0.016
POLAND	5	0.082	0.012	0.015
GREECE	1	3.52	0.012	0.014
IRELAND	5	1.392	0.012	0.014

### 3.4 Journal publications analysis

**Figure 5:**
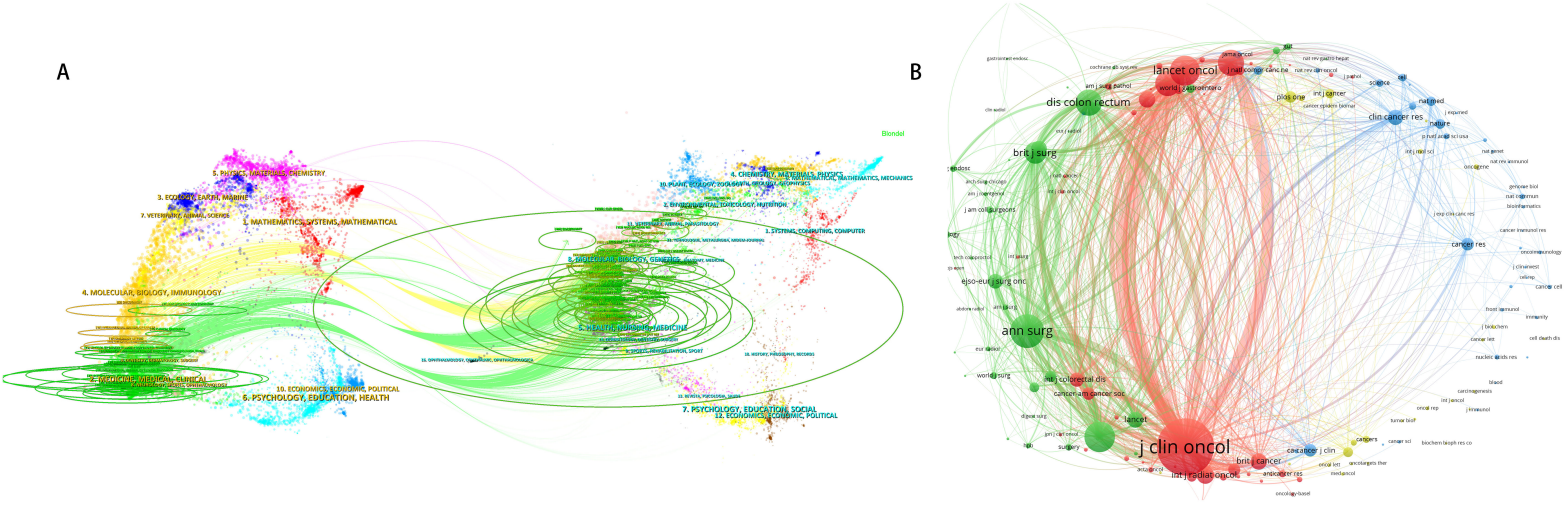
Neoadjuvant therapy **(A)** Dual map overlay. **(B)** Journal co-cited.

According to Bibliometrix analysis, the top journal in terms of the number of publications is Annals of Surgical Oncology Cancers. Through the dual map overlay utilizing CiteSpace, we employ citation link curves to connect the cited journals on both sides, with the length of the ellipses representing the number of authors and the width indicating the volume of publications. Our findings reveal that immunology, medical treatment, and clinical practice are influenced by genetics, health status, and nursing (**Figure 5A**). This suggests the existence of interdisciplinary intersections encompassing basic medical sciences, clinical medicine, nursing, and other social sciences within this domain.We further identified core journals in the field based on Bradford’s Law, which will help guide our literature search and article submission (**Supplementary Figure S1**). Co-cited journals show that in addition to journals with higher publication numbers, prestigious journal such as Lancet Oncology also has higher co-citation numbers (**Figure 5B**).

### 3.5 Analysis of co-citation reference and reference citation bursts

**Figure 6:**
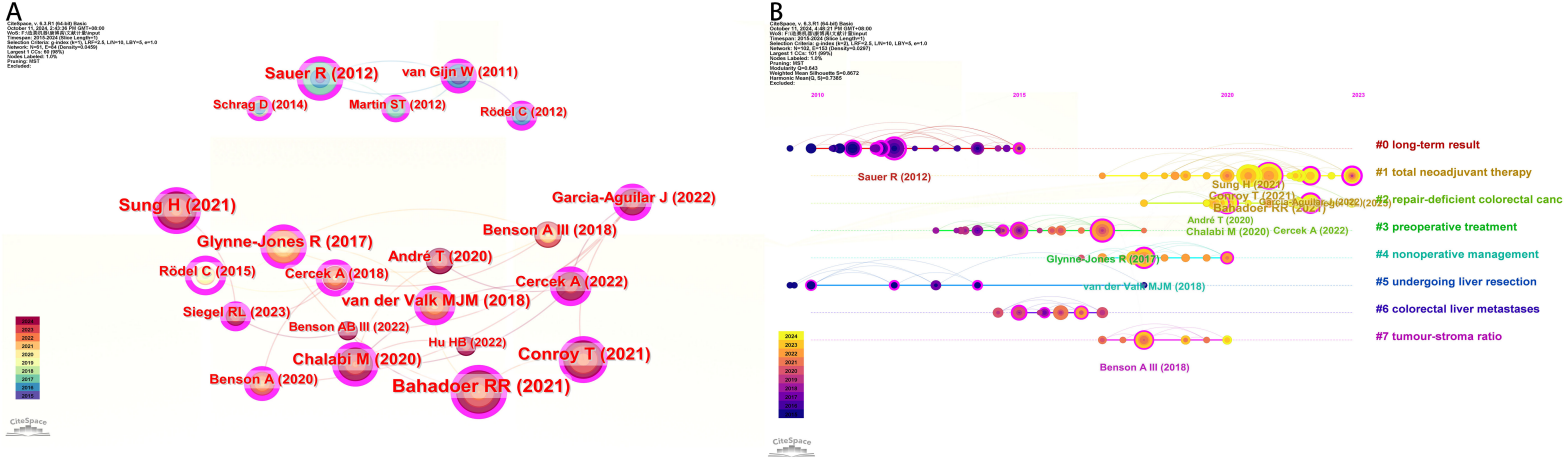
Reference analysis of neoadjuvant therapy. **(A)** Co-cited literature interaction. **(B)** Co cited literature label clustering analysis.

The analysis of co cited literature can help reveal research hotspots and frontiers in a field. In the literature co citation analysis graph, each node represents a different cited literature, and the line segments directly connected by the nodes indicate that they are cited in the same publication. We conducted co citation clustering analysis using CiteSpace and analyzed the top 20 cited literature (**Figure 6A**). Renu R. Bahadoor demonstrated in the article through multicenter, open label, randomized, phase III clinical trials that neoadjuvant chemotherapy has increased efficacy compared to adjuvant chemotherapy. This experimental treatment can be considered as a new nursing standard for high-risk locally advanced rectal cancer (10). Temporal analysis of co-cited literature categorizes the literature into eight types, with the position of the circle on the line indicating the temporal sequence of the literature (**Figure 6B**).

**Figure 7:**
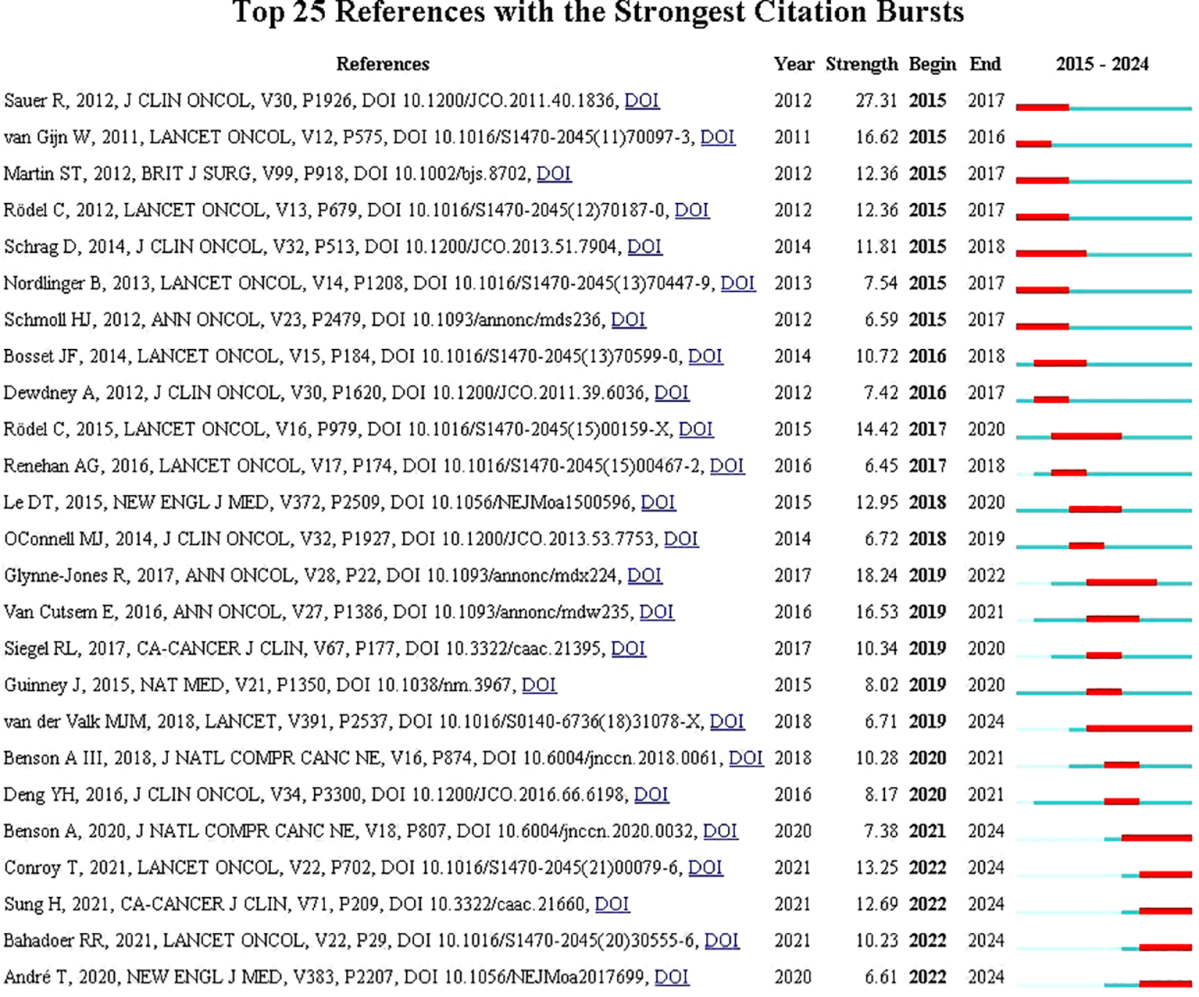
Burst analysis of co-citation literature.

Co-cited literature burst analysis is a high-frequency keyword that erupts at a specific moment, demonstrating the emergence of hotspots in the research area and indicating future research trends. The duration of the burst is displayed in red (**Figure 7**). The research results indicate that the research on neoadjuvant therapy for colorectal cancer was mostly published in the early 21st century, which suggests that the research in this field is relatively late and there is still much room for exploration.

### 3.6 Keyword co-occurrence and burst analysis

**Figure 8:**
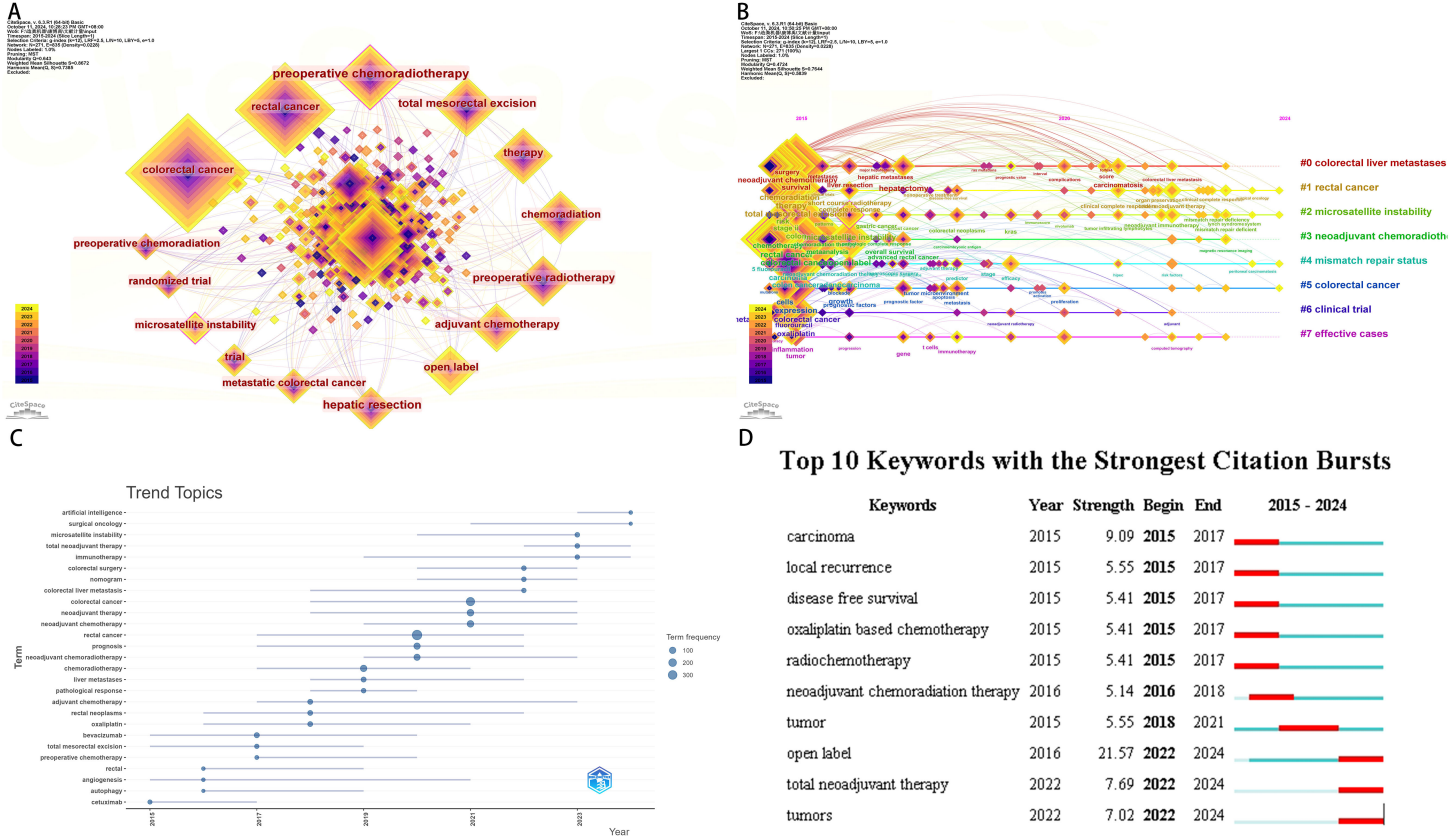
Neoadjuvant therapy for colorectal cancer. **(A)** Keyword co-occurrence **(B)** Keyword temporal analysis **(C)** Keyword trend topic **(D)** Keyword burst analysis.

Through keyword co-occurrence analysis, we isolated 270 keywords that appeared more than or equal to 3 times from the 2163 keywords contained in the dataset. Among them, “colorectal cancer” is the keyword with the most occurrences. In the keyword clustering analysis(**Figure 8A**), we clustered the keywords into 8 clusters and performed time series analysis. Combining time series analysis and trend topics, the timeline from 2015 to 2024 highlights the progress of keywords in this field. The analysis yields the following insights that in recent years, research in this field has mainly focused on “surgical oncology”. It also predicts that future research directions are anticipated to be associated to “artistic intelligence” (**Figure 8B, C**).

In conclusion, leveraging keyword co-occurrence analysis, keyword burst analysis, and temporal trend analysis, alongside insights from the Bibliometrix R package. This highlights the intricate nature of the neoadjuvant therapy landscape for colorectal cancer, which spans a diverse array of disciplines encompassing fundamental medical sciences, surgical specialties, internal medicine, pharmacology, nursing sciences, computer science, and social sciences alike. Consequently, there is a pressing need for interdisciplinary synergy and collaborative interventions within this complex research realm (**Figure 8D**).

## 4 Discussion

### 4.1 Summary of main findings

Through bibliometric analysis for visualizing literature, scholars can gain a fundamental understanding of a particular field, identify areas and directions of interest, and be encouraged to conduct further related research. More importantly, it allows us to delineate the historical and current research landscape of the field and predict future research directions. By utilizing the WoSCC for literature retrieval and downloading, we employed three distinct software packages—Bibliometrix R package, CiteSpace, and VOSviewer—to conduct both qualitative and quantitative analyses on the research outcomes in the field of neoadjuvant therapy for CRC over the past decade. These analyses covered various aspects including authors, countries, institutions, journals, co-cited literature, and keywords.

**Figure 9:**
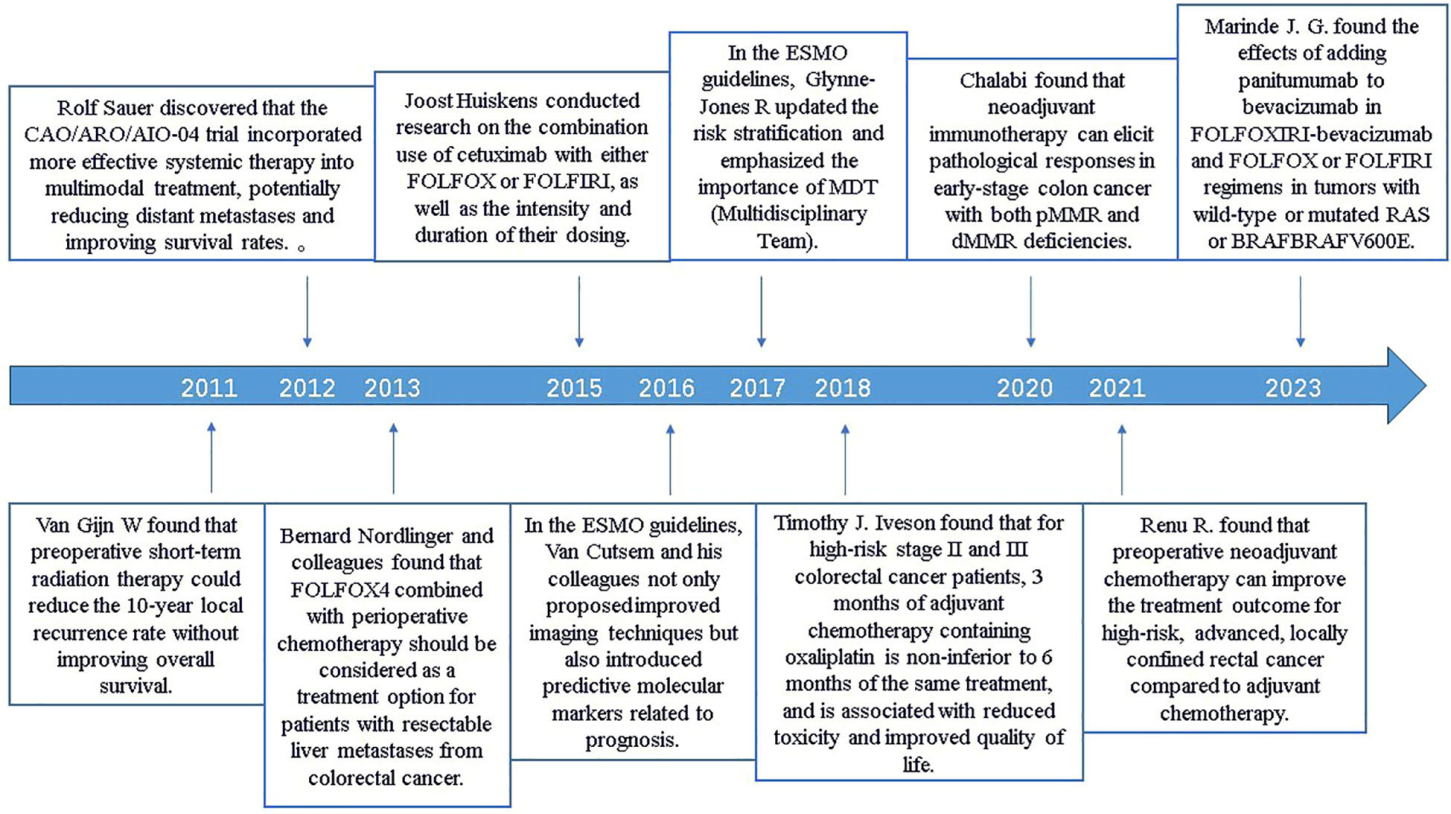
Important milestones in the neoadjuvant treatment of colorectal cancer.

As part of a treatment strategy, the implementation of neoadjuvant therapy presents both distinct advantages and challenges. It not only enables early reduction in tumor size and control of micrometastases but ensures options for organ preservation (11). Over the past decade, numerous scholars have conducted explorations and research in the field related to the treatment of CRC (12–14). We found that, after 2023, due to the introduction of standardized treatment protocols on one hand and a decrease in high-quality publications on the other, the average annual citation count in this field declined. However, it still maintains a high annual output of literature, suggesting that while continuing to pay attention to this field, we also need to improve the quality of publications. Our study emphasizes that German scholar Rolf Sauer is the most influential author in this field and the United States occupies a leading position in this field. Researchers can obtain the most advanced research results and ideas from American research institutions and academic conferences. China has significant development potential in the field of neoadjuvant therapy for CRC. The influence and status of the Netherlands cannot be ignored, which may potentially be linked to standard medical data management, an advanced clinical trial culture, and significant investments in medical research. Over the past decade, scholars have initially demonstrated the effectiveness of neoadjuvant therapy in patients with CRC, playing a pivotal role in promoting its adoption. Around 2016, through research on neoadjuvant therapy combined with chemotherapy, immunotherapy, and molecular markers, the efficacy of neoadjuvant therapy was further improved (15). By around 2020, scholars discovered the favorable outcomes of neoadjuvant therapy in rectal cancer, providing new insights for organ preservation and prognosis in rectal cancer patients (10). (**Figure 9**) We predict that future advancements in the field of colorectal cancer neoadjuvant therapy, particularly through the integration of artificial intelligence and neoadjuvant immunotherapy, will pave the way for even more significant advancements.

Through co-citation literature clustering and burst analysis, we have identified three primary research trends in this field.The foremost significance lies in the establishment of neoadjuvant treatment strategies for CRC. The latest Alliance A022104/NRG-GI010 trial in 2024, which aims to explore neoadjuvant treatment strategies to enhance organ preservation rates and improve the quality of life for locally advanced rectal cancer patients (16). The second major trend involves the exploration of neoadjuvant immunotherapy. The trend title indicates that neoadjuvant immunotherapy has garnered significant attention since the NICHE study in 2020. As a preferred option for patients with dMMR/MSI-H, it has improved overall survival and prognosis in patients with non-metastatic colon cancer, and this area of research will undoubtedly attract sustained attention in the forthcoming years (17). The third major trend concerns the identification of predictive biomarkers for neoadjuvant immunotherapy. An increasing amount of research is directed towards the investigation of predictive biomarkers. These evidences point towards the future trajectory of development in this direction.

### 4.2 Continuous updates in neoadjuvant treatment strategies for colorectal cancer

With the ongoing conduct of clinical trials, neoadjuvant treatment strategies for CRC continue to be updated. In the United States, extensive research has been conducted in the field of rectal cancer, with Phase II clinical trials including PICC, IMHOTEP, and NECTAR, and Phase III clinical trials comprising the UNION trial and UNICANCER-PRODIGE 23 (18–22). Additionally, the NEOPRISM study encompasses both Phase II and Phase III studies in a coherent manner (17). On one hand, these studies have explored the efficacy and safety of immunotherapy drugs such as pembrolizumab; on the other hand, they have also compared the advantages of long-course radiotherapy or short-course radiotherapy combined with immunotherapy. In terms of treatment strategies, the NCCN guidelines recommend the use of the FOLFIRINOX regimen only in patients with cT4N+, while FOLFOX or CAPEOX-based treatment regimens are considered for other scenarios. Based on the UNICANCER-PRODIGE 23 trial in 2021 and a small-sample prospective study in China, we suggest that the mFOLFIRINOX regimen can be used in stage III and IV CRC patients to improve their prognosis (22, 23). Currently, a study in South Korea is exploring the safety and feasibility of the mFOLFIRINOX regimen in high-risk stage III colon cancer patients (24). However, sufficient clinical evidence remains elusive. Some scholars have proposed innovative methods for delivering chemotherapy drugs through ultrasound targeted microbubble destruction (UTMD) to reduce treatment-related adverse reactions (25). Therefore, we hope that future research in this field can focus on new drug delivery methods to alleviate the pain caused by long-term intravenous injection and reduce drug damage to normal tissues. Besides chemoradiotherapy and immunotherapy, neoadjuvant targeted therapy has also become an option for patients with Her-2+ and Kras mutations. In terms of treatment duration, the clarification in these guidelines may be related to the results of the IDEA collaborative study in recent years around 2020 (26–29).

A vast number of scholars have conducted extensive explorations into neoadjuvant therapies for CRC. In the field of traditional Chinese medicine, quercetin (Qc) has pioneered a new herbal treatment paradigm for neoadjuvant therapy in CRC, offering additional therapeutic options (30). To address the recurrence of residual micrometastases, research teams have supplemented neoadjuvant therapy for CRC with a NIR-II photothermal and immunomodulatory integrated approach delivered through light-activated Mn2+ ions (31). There have also been updates in treatment strategies, with a new paradigm in CRC management called total neoadjuvant therapy (32). According to related studies in 2023, TNT, compared to traditional long-term and short-term neoadjuvant treatment regimens, has improved patient survival and reduced recurrence rates in high-risk CRC, while also facilitating organ preservation. Therefore, TNT stands as a favorable choice for patients with high-risk CRC and has the potential to expand indications beyond sphincter-preserving surgery for low rectal cancer (33, 34). In 2023, Japan conducted the TEGAFIRI trial, which explored the optimal strategy for TNT in locally advanced rectal cancer by using preoperative chemoradiotherapy with tegafur/uracil, oral calcium folinate, and irinotecan, followed by oxaliplatin-based chemotherapy as a TNT regimen (35). Therefore, the optimal neoadjuvant therapy strategy for CRC remains to be determined, and the current research challenge lies in balancing treatment efficacy and adverse events.

In clinical practice, surgery is generally performed 4-6 weeks after neoadjuvant therapy. However, the optimal interval between neoadjuvant therapy and surgery varies by region and remains controversial. A study from the Netherlands suggests that delaying surgery for 10-11 weeks after the completion of neoadjuvant chemoradiotherapy results in the highest likelihood of achieving a pathological complete response (pCR) in rectal cancer patients (36). However, results from a 2023 study indicate that patients with an interval of less than 8 weeks have a reduced chance of achieving pCR, while an interval greater than 12 weeks is associated with improved tumor regression grading (TRG) and a reduced risk of systemic recurrence (37). Longer intervals, however, can lead to more difficult surgical resection and a higher incidence of minor complications (38). In terms of treatment modalities, the Watch and Wait (W&W) strategy has opened up a new treatment paradigm for CRC patients, distinct from the traditional approach of surgery following neoadjuvant therapy. First proposed by Angelita Habr-Gama et al. in 2004 (39), W&W refers to patients, particularly those who achieve clinical complete response (cCR) after neoadjuvant chemoradiotherapy, who do not undergo traditional surgery but instead enter a period of close follow-up and observation, aiming to preserve organ function without compromising tumor survival rates. Following neoadjuvant therapy, one-third of patients can achieve a pCR (40). W&W has certain significance in eliminating the need for surgery and inpatient care to save costs (41). Advancements in neoadjuvant therapeutic strategies for CRC have facilitated global healthcare professionals in identifying efficient treatment modalities, thereby addressing, to a certain extent, the disparity in medical standards across various regions.

### 4.3 Neoadjuvant immunotherapy and predictive biomarkers related to immunotherapy for colorectal cancer may become a future research direction in this field

Although neoadjuvant chemoradiotherapy can reduce tumor stage, improve R0 resection rates, decrease local recurrence rates, and even achieve cCR or even pCR in some patients, it can also lead to postoperative complications such as anastomotic leakage, poor wound healing, sphincter function loss, and sexual dysfunction (42). Therefore, neoadjuvant immunotherapy emerges as a potential alternative.

The KEYNOTE-16 trial paved the way for neoadjuvant immunotherapy. Results from the NICHE I/II trials between 2020 and 2024 showed the efficacy and safety of neoadjuvant nivolumab with the addition of ipilimumab combination therapy in patients with locally advanced dMMR CRC, with a good pathological response (17, 43). However, in the previously mentioned KEYNOTE-177 study, nearly 30% of patients with dMMR/MSI-H advanced CRC did not respond to single-agent immunotherapy (44). Therefore, there is reason to believe that dual-agent neoadjuvant immunotherapy can be an option to improve efficacy. The VOLTAGE-A study was the first to explore the value of sequential chemoradiotherapy followed by immunotherapy in the neoadjuvant treatment of MSS rectal cancer, showing promising results (45).

Research on predictive biomarkers related to immunotherapy in CRC has also exerted a significant impact on this field. The use of immunohistochemical staining for MMR proteins and microsatellite analysis based on fluorescent multiplex PCR for patients with dMMR or MSI-H, followed by neoadjuvant immunotherapy, has emerged as a frontline treatment for unresectable metastatic CRC (46).

Many scholars have dedicated themselves to the research of other predictive biomarkers and have achieved certain results. A study in 2020 found that some MSS patients harbor POLE/POLD1 mutations and respond well to immunotherapy (47). High tumor mutational burden (TMB) is associated with immunotherapy, and several genes related to high TMB, including ARID1A, RNF43, BRAF, and KM2B in microsatellite instability (MSI) tumors, may also be used for the treatment of MSS patients (48). Classification based on consensus molecular subtypes (CMS) has also influenced the treatment of CRC patients, with CMS1 being considered more suitable for neoadjuvant immunotherapy (49). Low BRAFV600E mutation is also considered as an adverse predictive biomarker for advanced colon cancer (50). In CRC patients treated with immune checkpoint inhibitors, DNAH7 mutation predicts a better outcome (51). With the development of bioinformatics analysis, including artificial intelligence and deep learning, the use of deep learning-based classifiers has identified mutations in APC, KRAS, PIK3CA, SMAD4, and TP53 from H&E stained CRC pathology images as predictive of patient prognosis (52).

The tumor microenvironment is inhabited by various cell types, representing a heterogeneous but highly organized community (53). In terms of interferon signaling and antitumor immune populations, particularly the dense infiltration of CD8+ cytotoxic T lymphocytes, it predicts an increased likelihood of response to immune checkpoint inhibitors in CRC patients (54). The crucial role of the chemokine CXCL13 has been demonstrated in non-small cell lung cancer and esophageal squamous cell carcinoma, while its predictive role in neoadjuvant immunotherapy for CRC remains to be explored (55, 56). These explorations of the immune microenvironment enhance the prediction of treatment outcomes for CRC patients, aiding in the selection of appropriate treatment strategies for them.

## 5 Conclusion

This study conducted bibliometric analysis using CiteSpace, VOSviewer, and Bibliometrix R package, synthesizing the advantages of these three software packages to outline the research progress and frontier trends in neoadjuvant therapy for CRC globally. The newly published significant studies might be overlooked due to their lower citation counts. However, we still believe that this study has covered the research hotspots and future trends in neoadjuvant therapy for CRC, providing valuable information for relevant researchers.

In conclusion, this study may guide researchers in identifying directions for further research and provide important information and suggestions for those interested in this field, ultimately having a significant impact on the treatment of CRC patients.

## Data Availability

Original contributions are included in the article. Further inquiries may be directed to the corresponding authors.
