# Supplementary material for: Neoadjuvant therapy for colorectal cancer from 2015 to 2024: a visual analysis and bibliometric analysis
Source: Front Oncol. 2025 Apr 2;15:1526610. doi: 10.3389/fonc.2025.1526610 (PMC11999843; doi:10.3389/fonc.2025.1526610)
Supplement: Supplementary file 3 [file Table3.docx]

group1 <- c(1846,

44,

22,

22,

20,

14,

13,

11,

10,

9,

7,

5,

4,

4,

4,

4,

3

)

group2 <- c(848,

588,

536,

531,

435,

364,

340,

304,

299,

248,

154,

125,

116,

108,

102,

78,

73,

63,

57,

47,

42,

33,

30,

21,

21,

21,

20,

16,

15,

14,

12,

11,

9

)

result <- wilcox.test(group1, group2, exact=FALSE)

print(result)
