# Supplementary figures and images for: Neoadjuvant therapy for colorectal cancer from 2015 to 2024: a visual analysis and bibliometric analysis
Source: Front Oncol. 2025 Apr 2;15:1526610. doi: 10.3389/fonc.2025.1526610 (PMC11999843; doi:10.3389/fonc.2025.1526610)

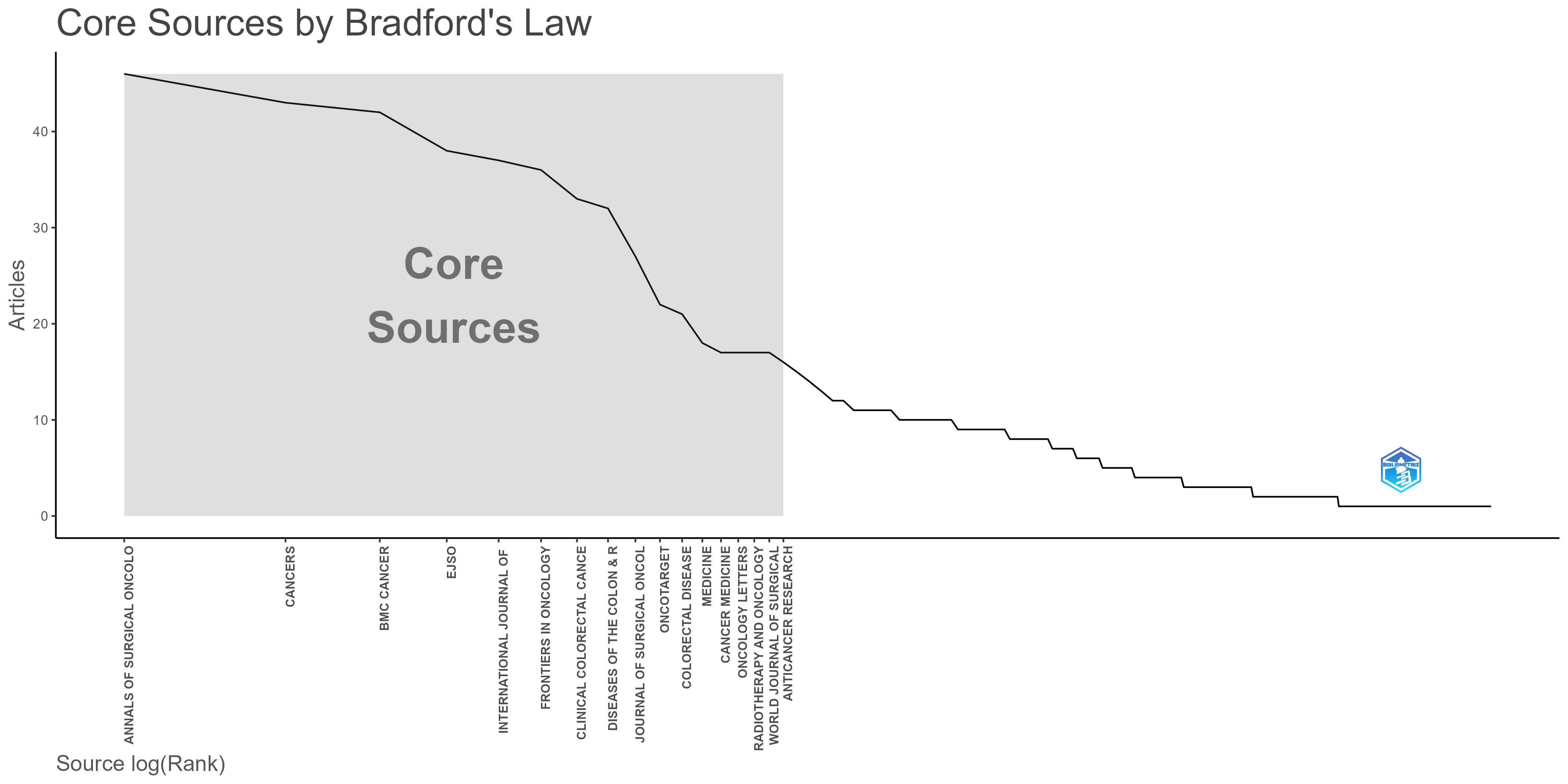

Supplement: Supplementary file 4 [file Image1.png]
